# An ancient haplotype containing antimicrobial peptide gene variants is associated with severe fungal skin disease in Persian cats

**DOI:** 10.1371/journal.pgen.1010062

**Published:** 2022-02-14

**Authors:** Alexandra N. Myers, Sara D. Lawhon, Alison B. Diesel, Charles W. Bradley, Aline Rodrigues Hoffmann, William J. Murphy, 99 Lives Cat Genome Consortium

**Affiliations:** 1 Department of Veterinary Pathobiology, College of Veterinary Medicine & Biomedical Sciences, Texas A&M University, College Station, Texas, Unites States of America; 2 Department of Small Animal Clinical Sciences, College of Veterinary Medicine & Biomedical Sciences, Texas A&M University, College Station, Texas, Unites States of America; 3 Department of Pathobiology, School of Veterinary Medicine, University of Pennsylvania, Philadelphia, Pennsylvania, Unites States of America; 4 Department of Veterinary Integrative Biosciences, College of Veterinary Medicine & Biomedical Sciences, Texas A&M University, College Station, Texas, Unites States of America; Clemson University, UNITED STATES

## Abstract

Dermatophytosis, also known as ringworm, is a contagious fungal skin disease affecting humans and animals worldwide. Persian cats exhibit severe forms of the disease more commonly than other breeds of cat, including other long-haired breeds. Certain types of severe dermatophytosis in humans are reportedly caused by monogenic inborn errors of immunity. The goal of this study was to identify genetic variants in Persian cats contributing to the phenotype of severe dermatophytosis. Whole-genome sequencing of case and control Persian cats followed by a genome-wide association study identified a highly divergent, disease-associated haplotype on chromosome F1 containing the S100 family of genes. *S100 calcium binding protein A9* (*S100A9*), which encodes a subunit of the antimicrobial heterodimer known as calprotectin, contained 13 nonsynonymous variants between cases and controls. Evolutionary analysis of *S100A9* haplotypes comparing cases, controls, and wild felids suggested the divergent disease-associated haplotype was likely introgressed into the domestic cat lineage and maintained via balancing selection. We demonstrated marked upregulation of calprotectin expression in the feline epidermis during dermatophytosis, suggesting involvement in disease pathogenesis. Given this divergent allele has been maintained in domestic cat and wildcat populations, this haplotype may have beneficial effects against other pathogens. The pathogen specificity of this altered protein should be investigated before attempting to reduce the allele frequency in the Persian cat breed. Further work is needed to clarify if severe Persian dermatophytosis is a monogenic disease or if hidden disease-susceptibility loci remain to be discovered. Consideration should be given to engineering antimicrobial peptides such as calprotectin for topical treatment of dermatophytosis in humans and animals.

## Author summary

Fungal skin infections known as ringworm or dermatophytosis affect billions of humans and animals worldwide. Normally the disease is self-limiting in affected individuals. The Persian cat breed is a popular breed known for its long hair coat and short nose as well as its propensity to develop severe, chronic dermatophytosis. By examining the genomes of Persian cats, we discovered that a specific region of DNA is highly altered between cats with and without severe dermatophytosis. The DNA sequence in this region is particularly divergent within a cluster of genes involved in immune defense against pathogens. Notably, alterations to the DNA sequence cause several changes in the antimicrobial protein known as calprotectin, which defends against pathogens in the skin of cats. Persian cats with severe dermatophytosis have a version of calprotectin similar to a version maintained by certain desert-dwelling wild felids such as sand cats and Asiatic wildcats. Therefore, we think this version of the protein is beneficial in some environments or against certain pathogens but not against the fungus that causes ringworm in cats. Our findings suggest changes to calprotectin may affect pathogen specificity and engineered calprotectin could be considered as a novel therapy for dermatophytosis in humans and animals.

## Introduction

Superficial fungal infections of the hair, skin, and nails affect an estimated one billion people globally and are the most common type of fungal infection in the world. These infections are most often caused by fungi known as dermatophytes, and the infection itself may be referred to as dermatophytosis, tinea, or a more commonly known name—ringworm [1]. While the disease is generally mild and self-limiting, its impact on human and animal welfare can be enormous [2]. A large, ongoing epidemic of treatment-resistant dermatophytosis in India has called attention to the serious economic and welfare implications of this disease and stimulated more research on pathogenesis and treatment [2–4].

Dermatophytes rely upon keratin as a source of nutrition. Ancestral dermatophytes gleaned this keratin primarily from soil, but recent adaptive radiation of this lineage has resulted in fungi that are adapted to keratins of specific host species [5–7]. Anthropophilic dermatophytes, such as those causing diseases commonly known as “athlete’s foot” and “jock itch”, are adapted to human keratins, while zoophilic dermatophytes are adapted to specific animal keratins [8]. *Microsporum canis* prefers the dog and cat as its primary host species but is commonly transmitted to humans [9].

Most dermatophyte infections are mild; however, severe and even fatal cases are occasionally reported [10,11]. In severe cases, the infection may become chronic, cover a large area of the body (extensive infection), and/or penetrate beyond the epidermis into the dermis and other tissues (deep infection). In humans, monogenic inborn errors of immunity can cause severe dermatophytosis, including autosomal recessive mutations in *caspase recruitment domain family member 9* (C*ARD9*) and autosomal dominant mutations in *signal transducer and activator of transcription 1* (*STAT1)* [11–14]. Reports of genetic variation contributing to disease susceptibility rather than severity also exist, although full functional validation of these variants is lacking [15,16].

**Fig 1:**
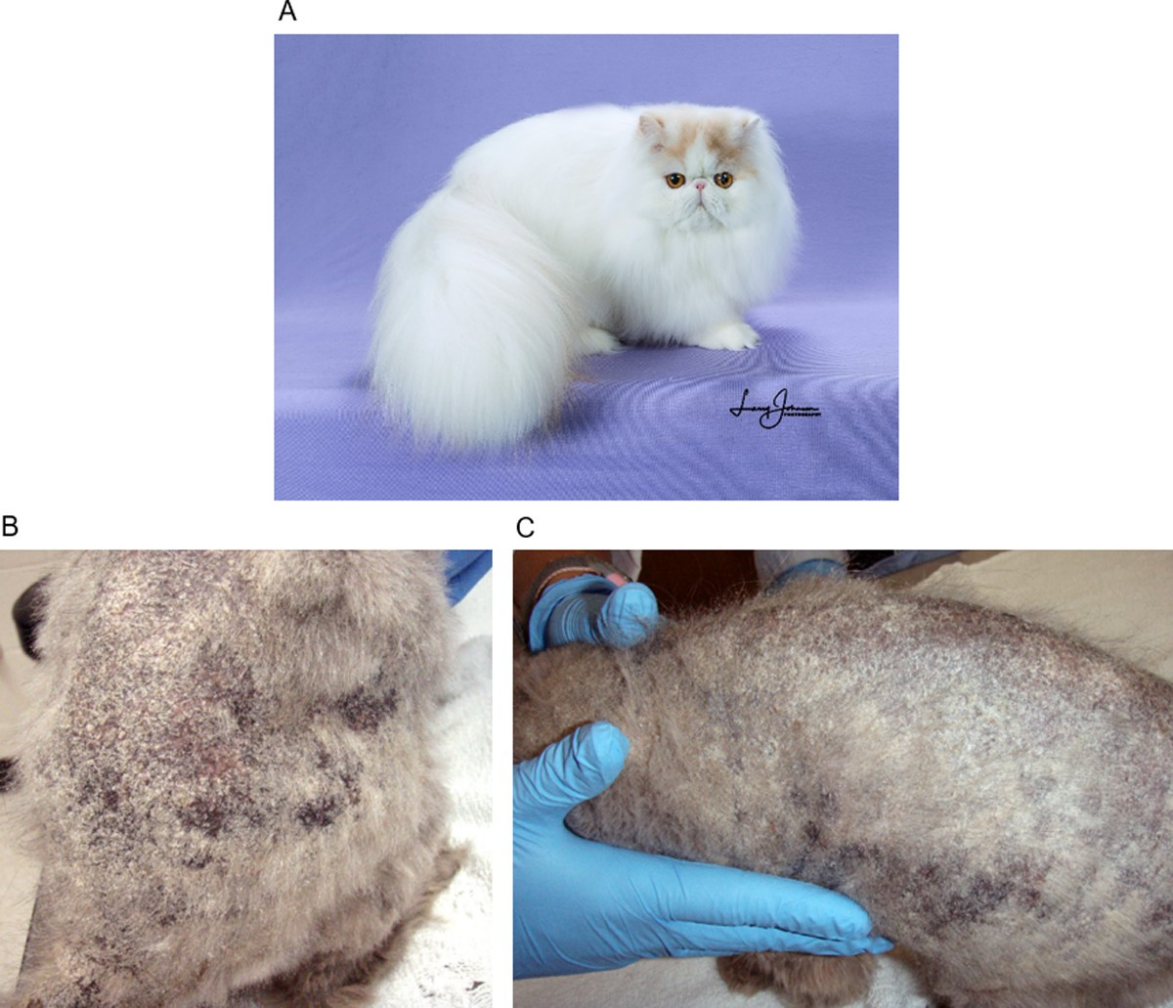
Macroscopic changes of a severe case of dermatophytosis in a Persian cat compared to a normal Persian cat. (A) A healthy, modern Persian cat with full hair coat. Photo courtesy of Larry Johnson. (B-C) Diffuse hair loss and scaling on the dorsum of a Persian cat with severe (chronic and extensive) dermatophytosis. This cat was not involved in the study.

Persian cats are more likely than other breeds to develop dermatophytosis caused by *M*. *canis*, including chronic, extensive, and/or deep infections (**Fig 1**) [9,17]. A unique form of deep dermal to subcutaneous dermatophytosis, termed dermatophytic pseudomycetoma, is seen almost exclusively in Persian cats. A genetic component to either susceptibility or severity of disease has long been suspected in this breed, supported by observations that particular Persian catteries housing genetically related cats have significantly higher prevalence of chronic disease than others [18]. A recessive mode of inheritance is suspected given severe, deep infections are relatively isolated to the Persian breed despite the Persian being used in the development of many other breeds of cat. Elucidating the genetic underpinnings of severe dermatophytosis in the Persian cat is likely to translate into better understanding of the pathogenesis and treatment of dermatophytosis in other species, including humans. The objective of this study was to identify genetic variants associated with severe dermatophytosis in Persian cats using whole-genome sequencing (WGS). We further investigated the expression and evolution of a key, skin-expressed antimicrobial peptide gene found within a divergent and disease-associated haplotype.

## Results

### A locus associated with severe dermatophytosis contains immune gene variants

**Fig 2:**
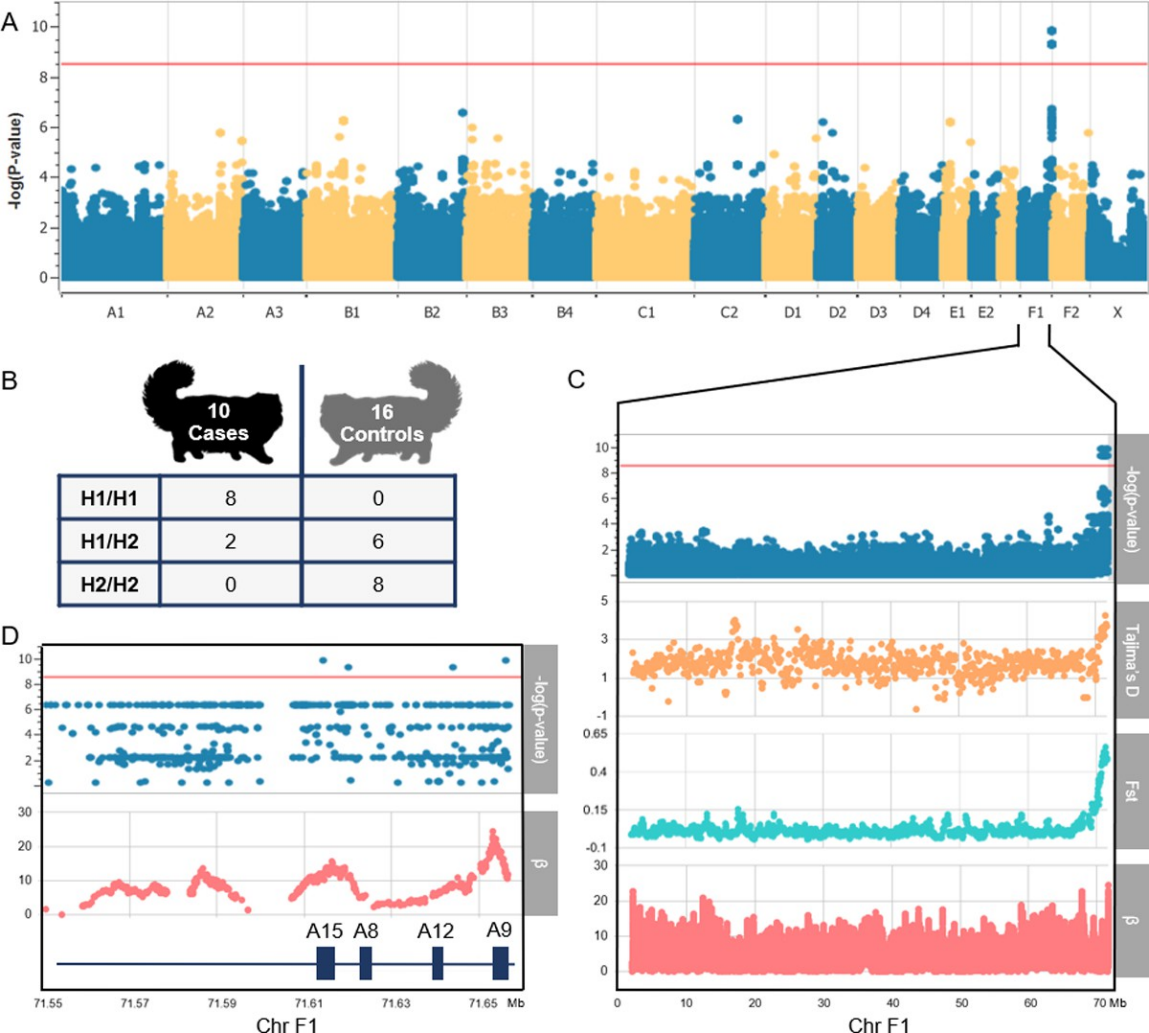
Genome-wide association analysis of Persians with and without severe dermatophytosis and evidence of divergent haplotypes under balancing selection. (A) Manhattan plot depicting single locus linear mixed model output for 10 Persian cat cases of severe dermatophytosis and 16 Persian cat controls. A locus on chromosome F1 achieves genome wide significance of p-value = 1.51x10^-10^. The Bonferroni-corrected significance threshold of p-value = 3.30 x 10^−9^ is shown in red. An additional Manhattan plot focused on the disease-associated locus can be found in S2 Fig. (B) Table depicting the genotypes of cases and controls for the disease-associated locus on F1. Two heterozygous controls are missing from the table as they were heterozygous for additional haplotypes (H3/H4 and H2/H6). (C) A Manhattan plot depicting the entirety of chromosome F1 is shown along with Tajima’s *D*, Weir and Cockerham’s Fst, and the β statistic for detection of long-term balancing selection. (D) A regional Manhattan plot depicting the last ~100 kb of chromosome F1 is shown along with β and the gene annotation. Within the disease associated locus, β is most elevated over the S100A antimicrobial peptide genes, *S100A9* (A9) and *S100A15* (A15).

All Persian cat whole genome sequencing data was uploaded to the NCBI Short Read Archive under BioProject PRJNA704916. Information on each Persian cat sampled for this study is listed in S1 **Table**. A genome-wide association study (GWAS) performed on variants called from the whole-genome sequencing data revealed a single peak of markers surpassing the Bonferroni correction threshold at the distal end of the acrocentric chromosome F1, with the most significant markers having a p-value of 1.51x10^-10^ (**Fig 2A**). A list of all significant markers and associated p-values can be found in S2 **Table**, and the GWAS quantile-quantile (QQ) plot and genomic inflation factor can be found in S1 **Fig**. Upon closer inspection, this peak marked the location of a large, ~1 Mb locus (chrF1:70,630,048–71,656,506) where eight of ten Persian cases (including the two cats with concurrent pseudomycetoma) were homozygous for a shared haplotype, henceforth referred to as domestic cat H1 (**Fig 2B)**. None of the 16 Persian control cats were homozygous for domestic cat H1, though 6 controls were heterozygous for this haplotype. This peak remained the only significant association whether the GWAS was conducted using all control cats (including those with unknown exposure to dermatophytes) or only the eight control cats with known previous exposure or mild/transient disease (S3 **Fig**). The ~1 Mb disease-associated locus contained 42 genes, eight of which contain disease-associated non-synonymous single nucleotide variants (SNVs) (**Tables 1, S3 and S4**). Half of these genes are well-characterized immune genes, including a cytokine receptor gene and antimicrobial peptide genes: *interleukin 6 receptor* (*IL6R)*, *S100 calcium binding protein A15 (S100A15)*, *S100A12*, and *S100A9*. The most significant non-synonymous SNV (p-value 1.51x10^-10^) is a missense mutation within *S100A9* predicted to cause a glycine to glutamic acid substitution: XP_003999832.3:p.Gly49Glu (S3 **Table**). Within the disease-associated locus, structural variant (SV) callers identified two deletions within the domestic cat H1 haplotype relative to the domestic cat H2 haplotype: a 1,321 bp intergenic deletion between *S100A6* and *S100A15* (F1:71,605,113) and a 255 bp intergenic SINE deletion between *S100A8* and *S100A9* (F1:71,648981). No SVs were found within coding regions or at the boundary of the disease-associated locus.

**Table 1:** Genes containing non-synonymous substitutions between case and control cats within the disease-associated locus.

Gene	Coding sequence length (bp)	Protein length (# amino acids)	# Non-synonymous SNPs	% identity between case and control protein	Function
*IL6R*	1155	385	1	99.7	Innate and adaptive immune response
*NUP210L*	5811	1937	4	99.8	Nuclear pore; RNA transport
*CREB3L4*	1269	423	1	99.8	Transcription factor
*CRTC2*	2094	698	2	99.9	cAMP response element binding
*S100A4*	399	133	1	99.2	Calcium, cAMP and lipid signaling
*S100A15*	324	108	1	99.1	Innate immune defense
*S100A12*	276	92	4	95.7	Innate immune defense
*S100A9*	405	135	13	90.4	Innate immune defense

Weir and Cockerham’s Fst and Tajima’s *D* were higher across the 1 Mb disease-associated locus than anywhere else on chromosome F1, indicating sequence divergence between cases and controls and an abundance of intermediate frequency alleles, respectively (**Fig 2C).** The beta statistic (β), a measure of long-term balancing selection, is also higher within the disease-associated locus than anywhere else on the chromosome (**Fig 2C**), but unlike Tajima’s D and Fst, the elevation is confined to a narrow region within the locus: the S100 antimicrobial peptide gene region (**Fig 2D)**. Together these findings suggest balancing selection is acting to maintain divergent haplotypes in this region.

### Divergent S100A9 haplotypes and comparison with wild felid haplotypes

**Fig 3:**
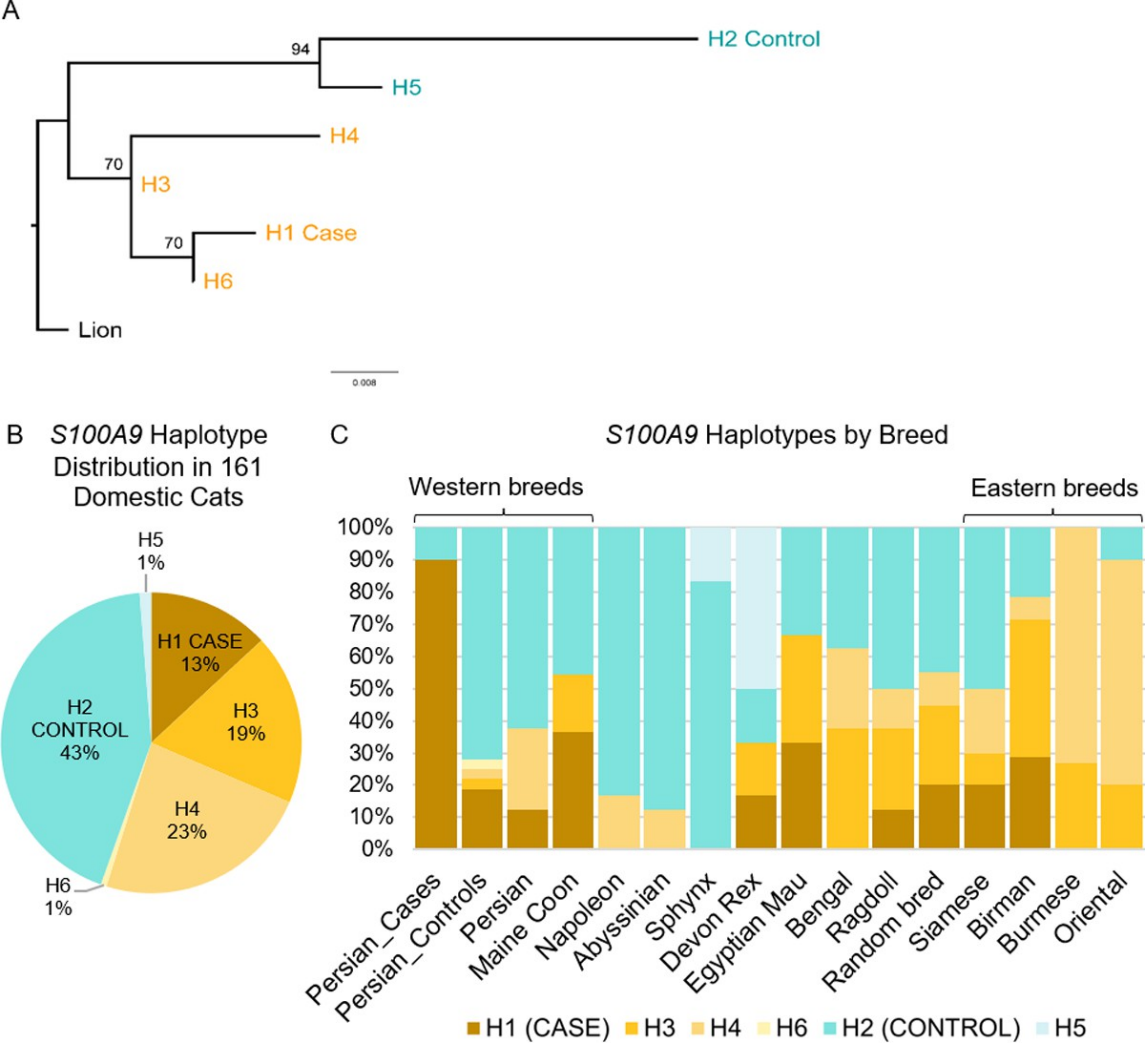
Haplotype frequencies of *S100A9* in the general cat population. (A) Maximum-likelihood phylogenetic tree inferred from the amino acid sequence of six distinct domestic cat *S100A9* haplotypes with the lion as the outgroup. Bootstrap support values are given on nodes. (B) Overall distribution of *S100A9* haplotypes in 161 domestic cats of varied breed and background. Haplotypes that cluster together with the Persian case haplotype (H1) are shaded in oranges while haplotypes that cluster with the Persian control haplotype (H2) are shaded in blues. (C) Relative frequency of haplotypes in various cat breeds. Labels for ‘Persian_Controls’ and ‘Persian_Cases’ represent the haplotype frequencies for the Persian cats enrolled in this study while the label for ‘Persian’ represents Persian cats in the general cat population. Haplotypes H3 and H4 predominate in cat breeds previously determined to be of Eastern origin. Breeds were included only if represented by at least 3 individuals (6 haplotypes).

**Fig 4:**
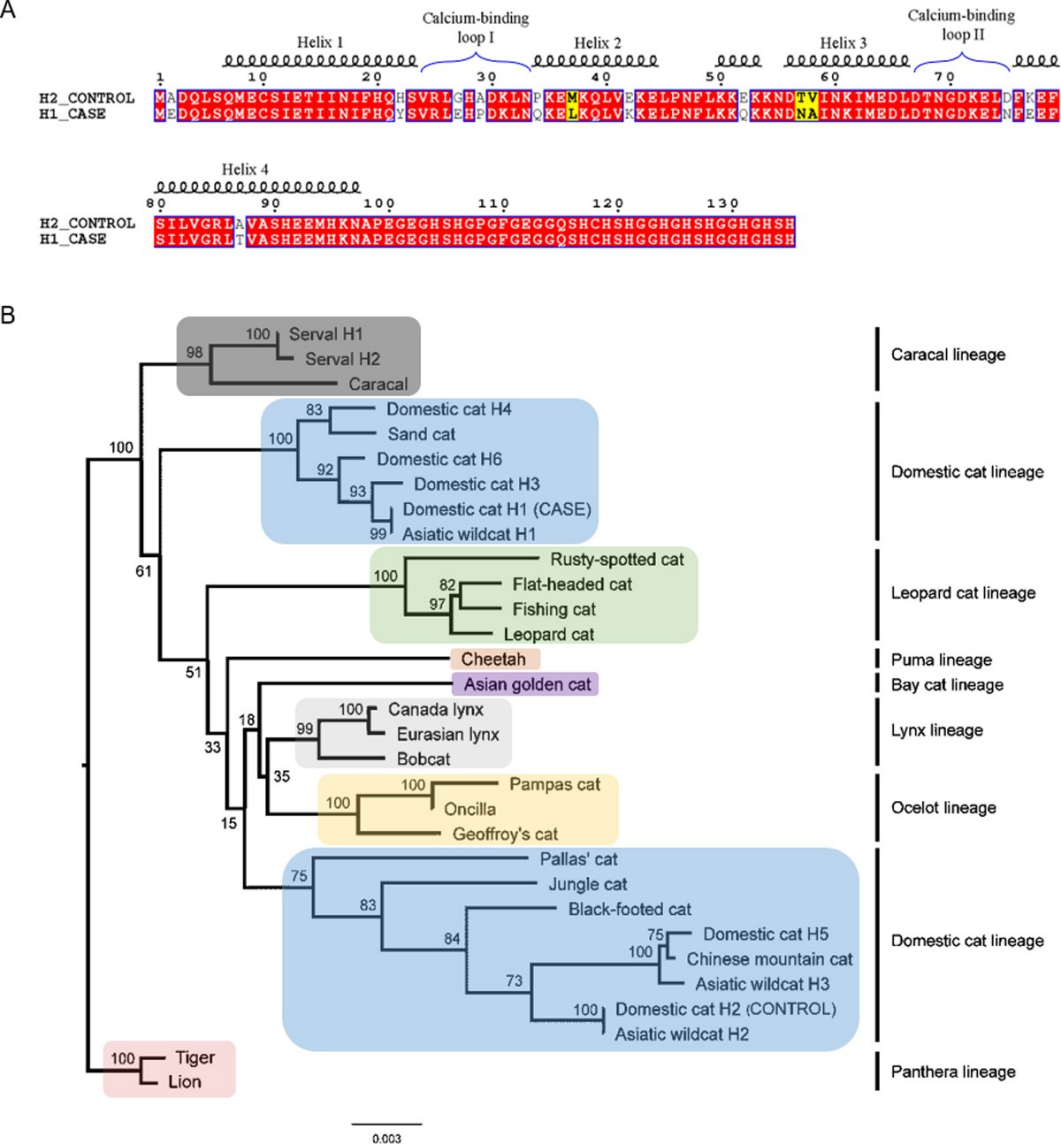
Highly divergent *S100A9* haplotypes and comparison with wild felids. (A) Amino acid sequence alignment and secondary structure depiction of the divergent Persian cat case and control *S100A9* haplotypes, created with ESPript 3.0. Conserved residues are boxed in red while similar residues are in bold text and boxed in yellow. (B) Maximum-likelihood phylogenetic tree inferred from the phased *S100A9* nucleotide sequences of 22 wild felids and the domestic cat haplotypes. Bootstrap support values are given on nodes. The domestic cat lineage is split across the tree: the Persian cat case haplotype (Domestic cat H1) clusters with the sand cat near the root of the tree while the control haplotype (Domestic cat H2) is found in the expected location for the domestic cat lineage.

Of all genes in the disease-associated locus, *S100A9* (F1:71,653,735–71,656,597) exhibited the highest sequence divergence between the common Persian case haplotype (domestic cat H1) and the common Persian control haplotype (domestic cat H2). Given this high level of sequence divergence, numerous amino acid alterations, its well-described role in antifungal immunity, and its reported epidermal expression in other species, *S100A9* was considered the most compelling candidate gene [19–21]. We therefore chose to focus on *S100A9* for further analysis. Upon examination of publicly available variant data from domestic cats of various breeds and backgrounds, six distinct *S100A9* haplotypes were identified in the domestic cat population, including domestic cat H1 and H2 as well as domestic cat H3, H4, H5, and H6 (**Fig 3A–3C**). Of note, the domestic cat H1 haplotype, which is homozygous in most Persian cases, had an allele frequency of 13% in the general domestic cat population, and 6.8% of this population are homozygous H1/H1. Both the domestic cat H1 and H2 haplotypes were also identified in a wild-caught Asiatic wildcat (*Felis silvestris ornata*) from Tajikistan. The domestic cat H1 and H2 *S100A9* haplotypes are highly divergent for two populations of the same species, sharing 97.91% nucleotide sequence identity across 2,864 base pairs and 90.4% identity across 135 amino acids. This protein, though relatively small, exhibited 13 amino acid substitutions between the common case (domestic cat H1) and control (domestic cat H2) haplotypes (**Figs 4A and S4**).

To explore the origin of the divergent alleles, a maximum-likelihood phylogenetic tree was inferred from the phased *S100A9* nucleotide sequences of 22 wild felids (publicly available sequence data) and six domestic cat *S100A9* haplotypes **(Fig 4B)**. The common Persian control haplotype (domestic cat H2) was found in its expected phylogenetic position with haplotypes from other species of the domestic cat lineage [22,23]. However, the Persian case haplotype (domestic cat H1) and the *Felis margarita* (sand cat) haplotypes formed a unique and highly divergent cluster from other *Felis* haplotypes as well as all other Felidae lineages. An approximately unbiased (AU) test confirmed this placement deviates significantly from the Felidae species tree topology (p-value = 6.19x10^-7^). While the domestic cat H1 *S100A9* nucleotide sequence was only 97.91% identical to the domestic cat H2 haplotype, it shared 99.27% identity with the sand cat sequence. The amino acid sequence of the domestic cat H1 haplotype shared 90.4% identity with the domestic cat H2 haplotype but 95.6% identity with the sand cat sequence. Overall, the Persian case haplotype is more diverged from the Persian control haplotype than from the sand cat haplotype [22]. Elsewhere in the phylogeny, there is little deviation from the species tree as would be expected with incomplete lineage sorting; therefore, ancient hybridization and introgression of this allele into ancestors of the sand cat and domestic cat populations is a more plausible explanation.

Alignment of raw reads from a case and control cat to a new, single-haplotype *Felis catus* genome assembly (BioProject ID PRJNA670214) revealed no differences in alignment or haplotypes from the standard felCat9 assembly (S5 **Fig**). Further, CNVnator analysis of chromosome F1 did not reveal gene duplications within the disease-associated locus. These findings indicate that genome assembly errors and copy number variation did not interfere with read mapping at this locus or lead to an artifactual increase in non-synonymous variation. Additionally, many cats are homozygous for the case or control haplotype, providing evidence that paralogous genes are not leading to mis-mapping of reads at this locus as this phenomenon should result in heterozygous sites.

### S100A8/S100A9 skin expression during feline dermatophytosis

**Fig 5:**
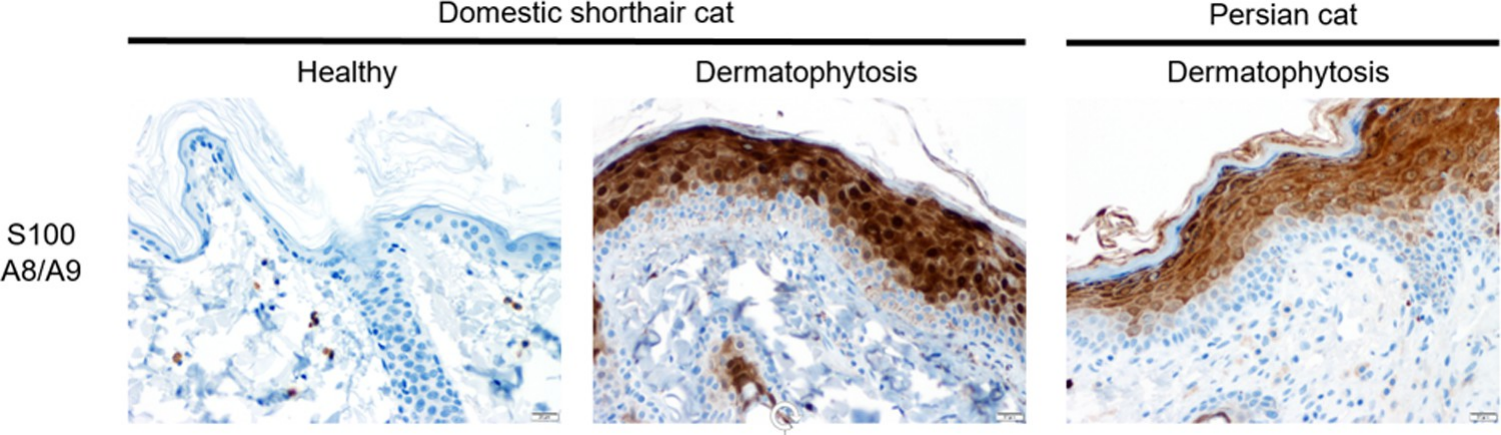
S100A8/S100A9 (calprotectin) immunohistochemistry performed on feline skin with and without dermatophytosis. No immunolabeling of the epidermis was observed in healthy domestic shorthair cats. Both domestic shorthair cats and Persian cats with dermatophytosis exhibited strong immunolabeling of the epidermis, excluding the basal cell layer. S100A8/S100A9 immunohistochemistry.

S100A9 and S100A8 dimerize *in vivo* to form the antimicrobial peptide known as calprotectin. Zero of five non-lesional domestic shorthair cats exhibited epidermal immunolabeling for S100A8/S100A9 (calprotectin) while ten of ten cats (5 domestic shorthair cats and 5 Persian cats) with dermatophytosis exhibited moderate to strong, multifocal to diffuse epidermal immunolabeling (**Fig 5**). These findings demonstrate a clear association between dermatophyte infection and increased expression of calprotectin in feline keratinocytes. This provides indirect evidence that calprotectin plays a role in defense against dermatophytes at the skin surface. We were unable to obtain sufficient concentrations of DNA from these FFPE skin samples to perform *S100A9* genotyping.

## Discussion

Using whole-genome sequencing, we identified a highly divergent haplotype containing antimicrobial peptide genes that was significantly associated with severe dermatophytosis in Persian cats. In particular, the *S100A9* gene encoding a subunit of the antimicrobial peptide (AMP) known as calprotectin included numerous amino acid substitutions between case (domestic cat H1) and control (domestic cat H2) haplotypes. We demonstrate that calprotectin (S100A8/A9) is likely to be an important player in anti-dermatophyte defense at the skin surface where this protein is highly expressed during infection.

Other genes within the disease-associated locus contained nonsynonymous mutations, but there are several reasons we chose to focus on *S100A9* as the most compelling candidate gene. First, the level of divergence between domestic cat H1 and H2 within *S100A9* was far higher than that of other genes with alteration of nearly 10% of amino acids between the haplotypes (13/135 amino acids). This striking level of divergence suggests some impact on function. Second, calprotectin has known antifungal activity and epidermal localization [19,24–26]. Other genes with multiple non-synonymous mutations such as *S100A12* and *NUP210L* are not reported to be skin-expressed; in humans, expression of *NUP210L* is reported to be almost exclusively testis-specific, making it an unlikely candidate gene [27]. As dermatophytes cause infection by adhering to and penetrating hair and epithelial cells at the skin surface, candidate genes for severe dermatophytosis are likely to be expressed in skin and/or hair. Certainly, mutations in other immune genes such as *S100A15* or *IL6R* also have the potential to play a role in severity and chronicity of infection and modulation of the immune response to various pathogens, as do non-coding mutations that may be impacting gene regulatory elements. The expression and regulation of multiple genes within this disease-associated locus are of interest and bear further investigation in the future.

AMPs such as calprotectin are an important component of the immune defenses of multicellular eukaryotes [28]. They are a highly diverse group of small proteins with activity against bacteria, fungi, and viruses [28]. Interestingly, AMPs were previously thought to have broad-spectrum activity against various pathogens; however, recent studies suggest a higher degree of specificity for particular pathogens and show that single amino acid substitutions can alter specificities [28]. The AMP calprotectin has been shown to exhibit activity against bacteria and fungi such as *Candida* sp. and *Aspergillus* sp., but studies evaluating its efficacy against dermatophytes are lacking [19,24–26]. Calprotectin exerts antimicrobial effects by chelating transition metals such as zinc and manganese needed for microbial growth—this is termed nutritional immunity [29]. Recently, however, Besold et al. demonstrated that calprotectin also inhibits bacterial growth through direct physical interaction that does not involve metal withholding [30]. The full mechanism for calprotectin’s antimicrobial action is still being elucidated.

Although the Persian cat case allele of calprotectin appears to render these cats susceptible to severe *Microsporum canis* infections, we suspect that this allele would be beneficial against other pathogens, given its relatively high frequency in the domestic cat population. AMPs of many species have been described to evolve under pathogen-driven balancing selection resulting from an evolutionary arms race between pathogens and the host immune proteins they directly interact with [31–34]. Maintenance of divergent alleles within a population can serve as a defense mechanism for a population by providing a heterozygote advantage and/or a reservoir of alleles that may be useful in specific conditions/environments or against particular pathogens [31]. Interestingly, high levels of non-synonymous variation have been previously identified within S100A antimicrobial peptide genes of taurine cattle (*Bos taurus*) and yak (*Bos grunniens*), suggesting a similar scenario of divergent haplotypes as observed in cats [35].

Previous studies evaluating signatures of selection in the Persian cat breed did not find evidence of selection on chromosome F1; however, these studies focused on selection signatures specific to the Persian breed, not other breeds or species [36,37]. We identified the divergent *S100A9* case and control alleles not only in various domestic cat breeds but also in an Asiatic wildcat (*Felis silvestris ornata*) from Tajikistan. Further, the case allele is similar to the sand cat (*Felis margarita*) allele, and together these alleles cluster much closer to the root of Felidae than expected. These findings indicate either introgression of the case allele into *Felis* species after an ancient hybridization event, long-term balancing selection, or a combination of the two (selection acting upon introgressed variation). We speculate that the haplotypes clustering in the clade with the sand cat and Persian case haplotypes may have been adaptive in the arid desert environments inhabited by the sand cat and many of the *Felis silvestris* subspecies thought to be the predecessors of modern domestic cats [38]. Arid regions harbor a unique repertoire of pathogens, and studies have shown dermatophytes are more prevalent in humid environments rather than arid conditions [39–41]. In modern long-haired Persian cats not confined to the desert, the divergent *S100A9* allele may be maladaptive. Further tests to assess effectiveness of the divergent calprotectin protein against other common feline pathogens should be undertaken to clarify any potential benefit of this allele.

Other explanations exist for why the case allele (domestic cat H1) is not as rare in the domestic cat population as might be expected (allele frequency of 13%) and why it is seen in breeds without a known predisposition to dermatophytosis. One explanation is that Persian cats possess a hidden disease susceptibility locus (or loci) not found in other breeds that we simply don’t have the power to detect with this study design. Alternately, it is possible that there are cases of severe dermatophytosis occurring in these other breeds that are not as well-recognized or reported. Clinical history regarding potential dermatophytosis is not readily available for the domestic cats in the publicly available dataset used here—some of the cats homozygous for H1/H1 in this dataset may have experienced severe disease. Additional work is suggested to characterize the frequency and severity of dermatophytosis in other cat breeds that carry domestic cat H1 or those haplotypes that cluster closely with it.

Regarding the two Persian cases that were not homozygous H1/H1 (both were H1/H2): we were unable to confirm that these cats had received adequate treatment for dermatophytosis, and they may have lived in highly contaminated environments. Indeed, one of these cats was the only cat included in the study known to have lived in a cattery with many other infected cats. Regardless of genotype, a highly contaminated environment may lead to chronic and eventually severe disease. Ideally, such environmental variables would be avoided in future studies by adhering to strict enrollment criteria including proper environmental decontamination.

The phenotype assessed in this study was that of chronic and extensive dermatophytosis in Persian cats despite appropriate veterinary-directed antifungal treatment. We were unable to specifically assess the phenotype of dermatophytic pseudomycetoma—a particular form of deep dermatophytosis that is also reported in humans—due to limited sample size [42,43]. Only two Persian cat cases included in this study had concurrent pseudomycetoma. These cats were homozygous for the case haplotype, but a larger sample size is needed. The clinical and histologic appearance of pseudomycetoma is quite different from that of superficial dermatophytosis and primarily involves infection of deeper layers of the skin and subcutaneous tissues rather than the epidermis; therefore, the immune genes and pathways involved may be different as well. Additional genotyping for pseudomycetoma cases is needed to determine if the *S100A9* case haplotype or closely-related haplotypes are associated with this specific phenotype. Overall, the small sample size of Persian cats enrolled for sequencing and GWAS was primarily due to our stringent enrollment criteria that 1) each Persian be diagnosed, sampled, and treated by a veterinarian (many owners and breeders attempt to diagnose and treat this disease on their own) and 2) that a detailed past history be known for case and control cats. However, several recent articles describe why small sample sizes can be used to successfully map disease loci in canine and feline GWAS [44,45]. In particular, dogs and cats have higher linkage disequilibrium than many other species due to inbreeding. Our findings here should still be validated and further explored in a larger cohort of cats while controlling for environmental variables as much as possible.

In summary, a divergent haplotype on chromosome F1 containing S100A antimicrobial peptide genes is associated with development of severe dermatophytosis in Persian cats. If this haplotype is not found to confer any significant benefit against other pathogens, a genetic test could be easily created to identify cats with the case haplotype and to help adjust breeding programs. These findings open the door for future investigation into AMPs as treatment options for dermatophytosis in humans and animals. Numerous AMPs are already in clinical trials for treatment of other diseases in humans [28,46,47]. Interest in engineering these peptides to increase specificity against particular pathogens is burgeoning, as AMPs are expected to have less trouble with development of antimicrobial resistance [48]. The application of certain forms of calprotectin as a topical treatment for dermatophytosis may be of value. Alternate treatments are becoming more important as the incidence of dermatophytosis continues to rise [41].

## Materials and methods

### Ethics statement

An animal use protocol (IACUC 2018–0256 CA) was approved by the Texas A&M University Institutional Animal Care and Use Committee to enroll clinical cases for this study.

### Sample collection and whole-genome sequencing

Study participants consisted of 26 adult Persian cats divided into ten cases of severe dermatophytosis and 16 controls (S1 **Table**). Severe dermatophytosis was diagnosed by a veterinarian in all cases via fungal culture at minimum, but typically multiple diagnostic tests were employed such as Wood’s lamp examination, microscopic examination of hair/skin, or PCR. Severe dermatophytosis was defined as chronic/recurrent infection covering a significant area of the body. In addition, two cases also had biopsy-confirmed dermatophytic pseudomycetoma, an uncommon form of deep dermatophytosis seen almost exclusively in Persian cats [49]. The remaining cases did not exhibit clinical signs of pseudomycetoma. Eight of ten cases had recurrence or continuation of infection despite receiving appropriate veterinarian-directed treatment; the remaining two cats could not be confirmed to have received appropriate treatment. Regarding the control cats, eight of 16 cats had documented past exposure to dermatophytes (at least six months prior to sample collection) but no history of severe or chronic infection. The remaining control cats had no history of dermatophytosis, but exposure status was unknown. Analyses were performed both with and without these cats of unknown exposure status.

Two buccal swabs (CytoSoft Cytology Brush, Medical Packaging Corporation, Camarillo, CA) were collected from each cat. DNA was extracted from buccal swabs within 1 week of sample collection using the salting-out method and Gentra Puregene reagents (Qiagen, Hilden, Germany). Standard paired-end Illumina libraries with 350 bp insert size were prepared for each cat using the NEBNext Ultra II FS DNA Library Prep Kit for Illumina and NEBNext Multiplex Oligos for Illumina (New England Biolabs, Ipswich, MA). Kits were used according to manufacturer guidelines. Libraries were sequenced to an average of 14X depth on the Illumina NovaSeq platform (Illumina, Inc., San Diego, CA).

### Variant calling and genome-wide association study

Data processing involved the following steps: 1) Quality and adapter trimming of raw reads with Cutadapt v1.18 [50], 2) alignment of reads to the feline reference genome (felCat9) using BWA-mem v0.7.17 [51], and 3) variant calling with GATK v4.1.0.0 following the Broad Institute’s best practices guidelines with the exception that hard filtering was performed rather than variant quality score recalibration [52]. Variant filtration thresholds as recommended by the Broad Institute were: quality by depth (QD) 2.0, Fisher strand (FS) 60.0, root mean square (RMS) mapping quality (MQ) 40.0, mapping quality rank sum test (MQ Rank Sum) −12.5, read position rank sum −8.0. Additional filtering was performed with VCFtools v0.1.16 using the following thresholds: quality 30, minimum allele frequency 5%, call rate 100%, and Hardy–Weinberg equilibrium (HWE) P > 1e−10. Only biallelic SNPs were retained. A final set of approximately 15.1 million SNPs was obtained after filtering. Structural variants were called with Manta v1.6.0 [53] and Delly v0.8.1 [54] in order to search for indels, inversions, or translocations that might disrupt coding sequence. Only variants called by both Manta and Delly were reported.

To correct for cryptic population structure and relatedness, a linear mixed model approach, EMMAX, was used for the GWAS in Golden Helix SNP & Variation Suite v8.8.3 (Golden Helix, Bozeman, MT) [55]. We used a Bonferroni-corrected genome-wide significance threshold of 3.30 x 10^−9^ based on the number of variants called in our data set. Population stratification was visualized with a QQ plot, and genomic inflation factor lambda (λ) was calculated as follows: λ = median (qchisq(1 –p, 1))/qchisq(0.5, 1) where p is the vector of GWAS *p*-values. The PROVEAN v1.1.3 web-based tool was used to classify the effect of variants on protein function (score <-2.5 indicates deleterious effect) [56]. Outside of the disease-associated locus, genes known to be involved in fungal immune pathways or skin barrier function were examined manually in IGV [57] for large deletions or other forms of copy number variation.

### Population genetics and evolutionary comparisons

First, steps were taken to eliminate copy number variation and genome assembly errors as a cause of increased non-synonymous variation at the disease-associated locus. Raw Illumina reads for a case and control cat were aligned to a new, single-haplotype *Felis catus* genome assembly (BioProject ID PRJNA670214) [58] to rule out felCat9 assembly errors (such as collapse of repetitive or highly divergent sequence) in the region of the disease-associated haplotype. Additionally, CNVnator was run for chromosome F1 to determine if sequence duplication could be contributing to increased sequence variation.

The sequence of *S100A9* was investigated among the general cat population using the 99 Lives Cat Genome Sequencing Consortium Database, which provides variant data for domestic cats of various breeds and backgrounds (**S6 Table**) [59,60]. *S100A9* haplotypes were identified from 191 cats via manual investigation of variant maps using Golden Helix SNP & Variation Suite v8.8.3 (Golden Helix, Bozeman, MT) (**S6 Table**). Haplotype frequencies were calculated overall and by breed. Only one cat from each bloodline was included in the final analysis when multiple cats were known to be related.

Domestic cat *S100A9* haplotypes were compared with *S100A9* sequences of 22 wild felids from the NCBI Short Read Archive (**S5 Table**). The publicly available Illumina sequence data from each of the 22 wild felids was trimmed and aligned to the domestic cat reference genome (felCat9) using BWA-mem. The resulting BAM files were examined in IGV. For each felid, a fasta file of the full *S100A9* nucleotide sequence was extracted using ANGSD v0.925 [61], and manual phasing was applied for those felids that had single nucleotide polymorphisms within this gene identified in IGV. Minimal phasing was needed as the majority of felids were homozygous or nearly homozygous for variants in this region. The phased wild felid and domestic cat haplotypes were aligned and a maximum likelihood tree was constructed using IQ-TREE v1.6.12 (-m GTR+I+G -b 500) [62]. The S100A9 nucleotide and amino acid alignments are found in **S1** and **S2 Data**, respectively. An approximately unbiased (AU) test was performed using IQ-TREE to determine if the *S100A9* gene tree was significantly different from the Felidae species-tree topology (inferred from low-recombining regions of the X-chromosome by Li et al.) [23].

To examine forces of natural selection acting upon *S100A9*, we evaluated Weir and Cockerham’s Fst and Tajima’s *D* for the Persian cats included in this study using VCFtools v0.1.16. We evaluated Tajima’s *D* in 100 kb windows and Fst in 100 kb sliding windows across chromosome F1. We additionally calculated the beta statistic (β), designed to detect ancient balancing selection using BetaScan (default settings with the addition of -w 6000 and -fold options) [63]. Repetitive regions and regions exhibiting copy number duplication, as identified by WindowMasker and CNVnator respectively, were masked for this analysis [64,65].

### Immunohistochemistry

To assess and localize S100A8/S100A9 (calprotectin) expression in normal and affected feline skin, immunohistochemistry (IHC) was performed on archived FFPE cat skin using an antibody to human calprotectin that has been used extensively in various species including cats [66,67]. IHC was performed on skin from five healthy domestic shorthair cats, five domestic shorthair cats with dermatophytosis, and five Persian cats with dermatophytosis. Attempts were made to extract DNA from these FFPE skin samples for PCR-based genotyping of the S100A9 region using the BiOstic FFPE Tissue DNA Isolation Kit (MoBio Laboratories, Inc., Carlsbad, CA, USA); however, these samples did not yield sufficient quality DNA for accurate genotyping.

FFPE sections of feline skin were cut at 4 μm and mounted on charged slides. The sections were deparaffinized in xylene and rehydrated through graded alcohols. Antigen retrieval was performed by immersing the slides in a citrate buffer and heating them in a pressure cooker (Decloaking Chamber, Biocare Medical, Pacheco, CA). After retrieval, the slides were washed with tris buffer prior to beginning the immunostaining procedure. The immunohistochemical procedure was run on an automated platform (intelliPATH FLX, Biocare Medical). All incubations were conducted at room temperature. Endogenous peroxide activity was blocked by incubating the slides with hydrogen peroxide for 10 minutes. The sections were then incubated with the primary antibody (mouse anti-human Myeloid/Histiocyte Antigen, clone MAC 387, dilution 1:200, Agilent Technologies, Santa Clara, CA) for 30 minutes. Next, the slides were incubated with a polymer detection reagent (Mouse-on-Canine HRP Polymer, Biocare Medical) for 40 minutes. Sites of antibody-antigen interaction were visualized with a DAB chromogen (ImmPACT DAB Substrate kit, peroxidase, Vector Laboratories, Burlingame, CA). The sections were then counterstained with Mayer’s hematoxylin. A negative control reagent (Universal Negative Control Serum, Biocare Medical) was substituted for the primary antibody for the negative control tissues. Each sample was assessed for intensity and distribution of keratinocyte staining.

## Supporting information

S1 Fig Quantile-Quantile plot for the genome-wide association analysis of Persians with and without severe dermatophytosis, including all cases and controls. (PDF)

S2 Fig Additional Manhattan plot of the genome-wide association analysis of Persians with and without severe dermatophytosis. Scaling has been adjusted to show the entire ~1 Mb disease-associated locus on chromosome F1. As in Fig 2B, the Manhattan plot depicts single locus linear mixed model output for 10 Persian cat cases of severe dermatophytosis and 16 Persian cat controls. The grey block at the right of the image represents the end of chromosome F1. The Bonferroni-corrected significance threshold of p-value = 3.30 x 10–9 is shown in red.
(PDF)

S3 Fig Genome-wide association analysis of Persians with and without severe dermatophytosis, including only those control cats with prior confirmed exposure to dermatophytes. Manhattan plot depicting single locus linear mixed model (EMMAX) output for 10 Persian cat cases of severe dermatophytosis and 8 Persian cat controls that were previously exposed to dermatophytes without developing severe disease. As in the GWAS performed with all 16 control cats, a single peak of SNPs on chromosome F1 surpasses the Bonferroni threshold.
(PDF)

S4 Fig S100A9 amino acid alignment for wild felids and domestic cat haplotypes. (PDF)

S5 Fig IGV output of raw reads from a Persian case and a Persian control aligned to a single-haplotype Felis catus genome assembly (BioProject ID PRJNA670214). This single-haplotype genome has the Persian case haplotype (H1) at S100A9, hence the lack of SNVs in the Persian case alignment. The Persian control cat is homozygous for the control haplotype (H2). Marked variation between the case and control haplotypes is apparent.
(PDF)

S1 Table List of all Persian cats sampled for whole-genome sequencing and genome-wide association study. Known related pairs of cats (parents or siblings) are indicated with matching colors.
(PDF)

S2 Table List of all SNPs surpassing the Bonferroni correction threshold and their associated p-values from genome-wide association analysis of Persians with and without severe dermatophytosis. (PDF)

S3 Table All non-synonymous substitutions between the most common case haplotype (H1) and the most common control haplotype (H2) within the disease-associated locus. (PDF)

S4 Table List of all 42 genes within the disease-associated locus and status of feline skin expression based on publicly available feline RNA-seq data. (PDF)

S5 Table NCBI accession numbers of wild felid sequences used to create S100A9 nucleotide and amino acid alignments. (PDF)

S6 Table S100A9 haplotypes of 191 domestic cats from the 99 Lives Cat Genome Sequencing Initiative (NCBI accession #PRJNA308208). (XLSX)

S1 Data S100A9 phased nucleotide alignment for domestic and wild felids. (FAS)

S2 Data S100A9 phased amino acid alignment for domestic and wild felids. (FAS)
