# Supplementary figures and images for: An ancient haplotype containing antimicrobial peptide gene variants is associated with severe fungal skin disease in Persian cats
Source: PLoS Genet. 2022 Feb 14;18(2):e1010062. doi: 10.1371/journal.pgen.1010062 (PMC8880935; doi:10.1371/journal.pgen.1010062)

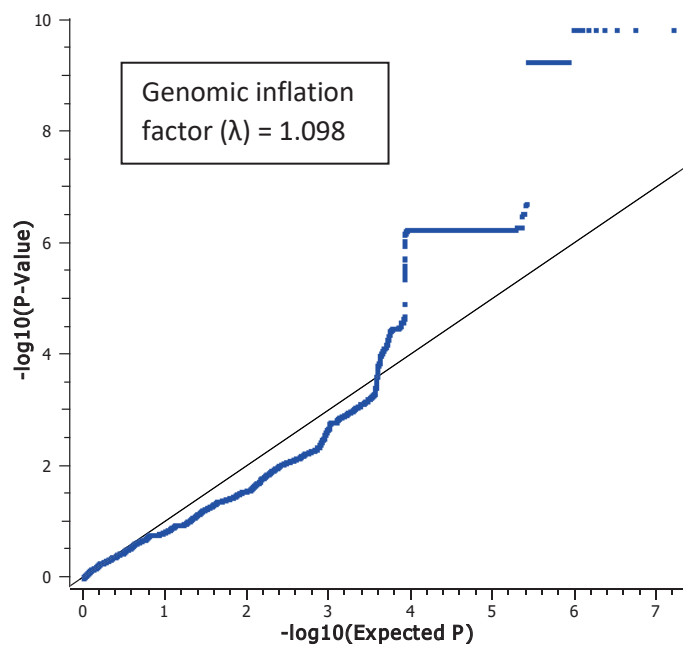

Supplement: S1 Fig — (PDF) [file pgen.1010062.s001.pdf]

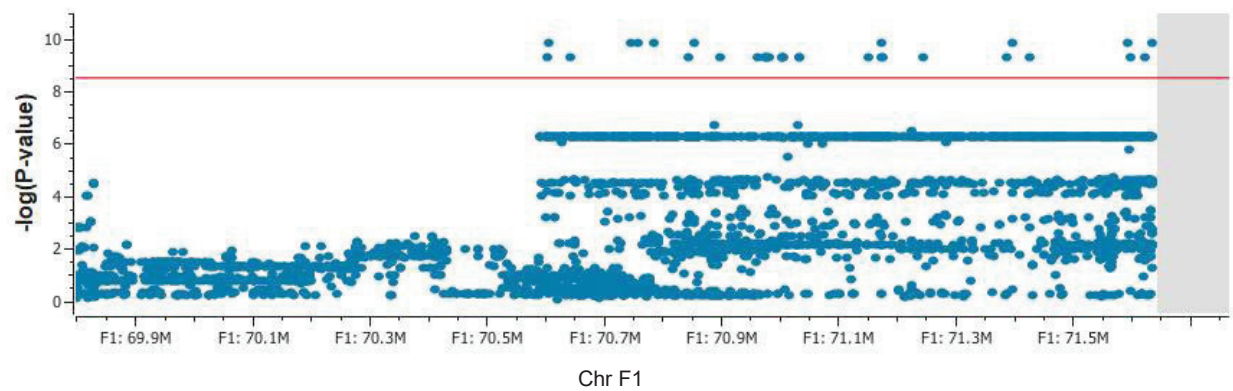

Supplement: S2 Fig — Scaling has been adjusted to show the entire ~1 Mb disease-associated locus on chromosome F1. As in Fig 2B, the Manhattan plot depicts single locus linear mixed model output for 10 Persian cat cases of severe dermatophytosis and 16 Persian cat controls. The grey block at the right of the image represents the end of chromosome F1. The Bonferroni-corrected significance threshold of p-value = 3.30 x 10^–9^ is shown in red. (PDF) [file pgen.1010062.s002.pdf]

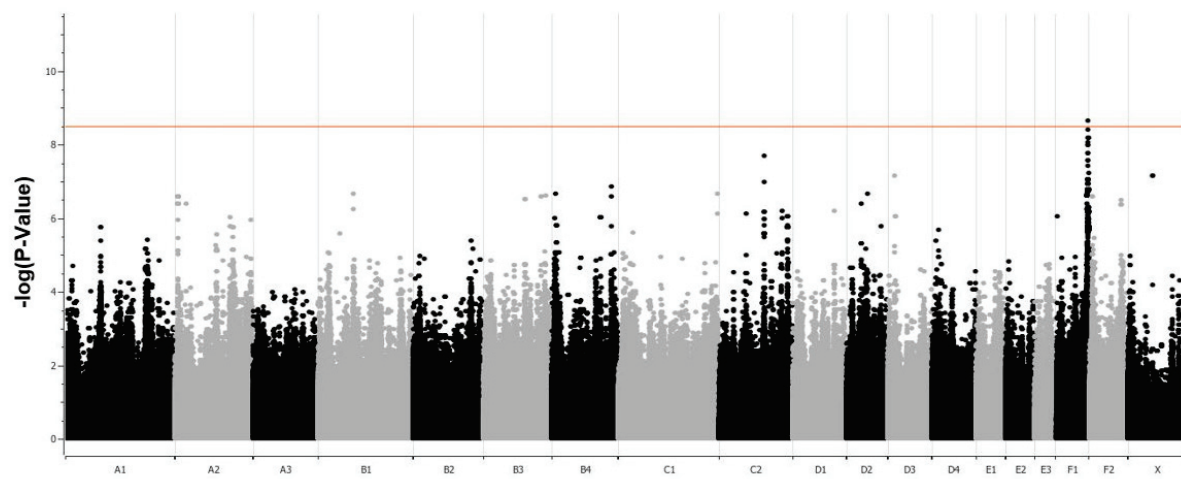

Supplement: S3 Fig — Manhattan plot depicting single locus linear mixed model (EMMAX) output for 10 Persian cat cases of severe dermatophytosis and 8 Persian cat controls that were previously exposed to dermatophytes without developing severe disease. As in the GWAS performed with all 16 control cats, a single peak of SNPs on chromosome F1 surpasses the Bonferroni threshold. (PDF) [file pgen.1010062.s003.pdf]

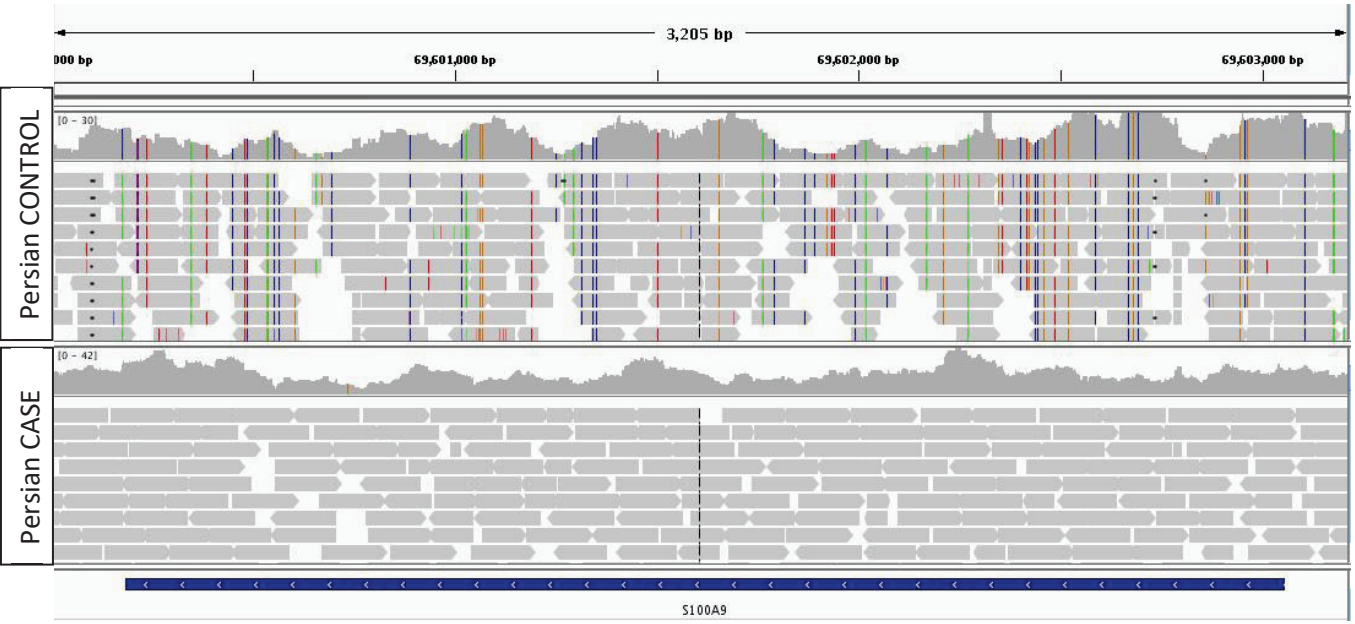

Supplement: S5 Fig — This single-haplotype genome has the Persian case haplotype (H1) at S100A9, hence the lack of SNVs in the Persian case alignment. The Persian control cat is homozygous for the control haplotype (H2). Marked variation between the case and control haplotypes is apparent. (PDF) [file pgen.1010062.s005.pdf]
