# Supplementary material for: An ancient haplotype containing antimicrobial peptide gene variants is associated with severe fungal skin disease in Persian cats
Source: PLoS Genet. 2022 Feb 14;18(2):e1010062. doi: 10.1371/journal.pgen.1010062 (PMC8880935; doi:10.1371/journal.pgen.1010062)
Supplement: S4 Fig — (PDF) [file pgen.1010062.s004.pdf]

```
 1 Domestic_cat_H2_(CONTROL) MADQLSQMECSIETIINIFHQHSVRLGHADKLNPKEMKQLVEKELPNFLKKEKKNDTVINKIMEDLDTNGDKELDFKEFSILVGRLAVASHEEMHKNAPEGEGHSHGPGFGEGGQSHCHS
 2 Asiatic_wildcat_H2        ........................................................................................................................
 3 Black-footed_cat          .....................Y...........Q.......K...L..........N...............................................................
 4 Jungle_cat                .....................YA.Q...P....Q....K..K...L..........N............V..................................................
 5 Asiatic_wildcat_H3        ..........C..........Y.L.........Q.......K...L.....Q....NA..............................................................
 6 Domestic_cat_H5           .....................Y...........Q.......K...L.....Q....NA..............................................................
 7 Chinese_mountain_cat      .....................Y...........Q.......K...L.....Q....NA..............................................................
 8 Pallas_cat                .....................YA.W.K.Q....Q.......K.............GNA..................E...........................................
 9 Cheetah                   .....................Y....P.P....H.......K.........Q....NA..................E.........T.........................S.......
10 Canada_lynx               .....................Y......P....Q.......K.........Q....NA..................E......A..T.................................
11 Eurasian_lynx             .....................Y......P....Q.......K.........Q....NA..................E......A..T.................................
12 Bobcat                    .....................Y......P....Q.......K.........Q....NA............................T...............................Q.
13 Pampas_cat                .....................Y......P....Q.......K.........Q....NA..................E.........T.................................
14 Oncilla                   .....................Y......P....Q.......K.........Q....NA..................E.........T.................................
15 Geoffroys_cat             .....................Y......P....Q.......K.........H....NA..................E.........T.................................
16 Asian_golden_cat          .....................Y......P.Q..Q.......K.........Q....NA............................T.................................
17 Sand_cat                  ...K..........V......Y......P....LE.L....K.........Q....NA................N.E.........T.................................
18 Domestic_cat_H1_(CASE)    .E...................Y....E.P....Q..L....K.........Q....NA................N.E.........T.................................
19 Asiatic_wildcat_H1        .E...................Y....E.P....Q..L....K.........Q....NA................N.E.........T.................................
20 Domestic_cat_H3           .....................Y......P....Q..L....K.........Q....NA................N.E.........T.................................
21 Domestic_cat_H4           .....................YC.....P.....E.L....K.........Q....NA................N.E.........T.................................
22 Serval_H1                 .....................Y......P....Q.......K.........Q....NA................N.E.........T.................................
23 Serval_H2                 .....................Y..Q.A.P....Q.......K.........Q....NA................N.E.........T.................................
24 Caracal                   .....................Y......P....Q..L....K.........Q....NA................N.E..T......T.................................
25 Rusty-spotted_cat         ...........L.........YA.....P....Q..L....K...S.....Q....NA................N.E.........T.................................
26 Flat-headed_cat           .....................Y......P.Q..Q.......K.........Q....NA..................N.........T.................................
27 Fishing_cat               .....................Y......P.Q..Q.......K.........Q....NA..................N.........T.................................
28 Leopard_cat               .....................Y......P....Q.......K..............NA...T...........V..N.........T.................................
29 Tiger                     .....................Y......P....Q.......K.........Q....NA................N.E.........T.................................
30 Lion                      .....................Y......P....Q.......K.........Q....NA................N.E.........T...Q............................

 1 Domestic_cat_H2_(CONTROL) HGGHGHSHGGHGHSH*
 2 Asiatic_wildcat_H2        ...............*
 3 Black-footed_cat          ...............*
 4 Jungle_cat                ...............*
 5 Asiatic_wildcat_H3        ...............*
 6 Domestic_cat_H5           ...............*
 7 Chinese_mountain_cat      ...............*
 8 Pallas_cat                ...............*
 9 Cheetah                   ...............*
10 Canada_lynx               ...............*
11 Eurasian_lynx             ........C......*
12 Bobcat                    ...............*
13 Pampas_cat                ...............*
14 Oncilla                   ...............*
15 Geoffroys_cat             ...............*
16 Asian_golden_cat          ...............*
17 Sand_cat                  ...............*
18 Domestic_cat_H1_(CASE)    ...............*
19 Asiatic_wildcat_H1        ...............*
20 Domestic_cat_H3           ...............*
21 Domestic_cat_H4           ...............*
22 Serval_H1                 .S.............*
23 Serval_H2                 .S.............*
24 Caracal                   .S.............*
25 Rusty-spotted_cat         .S.............*
26 Flat-headed_cat           .S.............*
27 Fishing_cat               .S.............*
28 Leopard_cat               ...............*
29 Tiger                     ...............*
30 Lion                      ...............*
```
