# Supplementary material for: An ancient haplotype containing antimicrobial peptide gene variants is associated with severe fungal skin disease in Persian cats
Source: PLoS Genet. 2022 Feb 14;18(2):e1010062. doi: 10.1371/journal.pgen.1010062 (PMC8880935; doi:10.1371/journal.pgen.1010062)
Supplement: S1 Table — Known related pairs of cats (parents or siblings) are indicated with matching colors. (PDF) [file pgen.1010062.s006.pdf]

S1 Table

Cat #	Case/Control	Age (years)	Sex	# Ringworm Episodes	Pseudomycetoma	*S100A9* Haplotype
1	control	13	spayed female	0	no	H2/H2
2	control	11	spayed female	0	no	H2/H2
3	control	5	castrated male	0	no	H1/H2
4	control	14	spayed female	0	no	H2/H2
5	control	6	intact male	0	no	H2/H2
6	control	4	intact male	0	no	H1/H2
7	control	13	castrated male	0	no	H1/H2
8	control	12	spayed female	0	no	H1/H2
9	control	5	castrated male	1 mild	no	H1/H2
10	control	8	castrated male	1 mild	no	H2/H2
11	control	11	castrated male	1 mild	no	H2/H2
12	control	12	spayed female	1 mild	no	H2/H2
13	control	11	castrated male	1 mild	no	H2/H2
14	control	10	castrated male	0 but exposed	no	H2/H6
15	control	10	spayed female	0 but exposed	no	H1/H2
16	control	4	intact female	1 mild	no	H3/H4
17	case	9	spayed female	>1 severe	no	H1/H1
18	case	13	castrated male	>1 severe	no	H1/H1
19	case	2	castrated male	>1 severe	no	H1/H1
20	case	12	intact male	>1 severe	no	H1/H1
21	case	12	intact male	>1 severe	no	H1/H1
22	case	9	intact female	>1 severe	no	H1/H1
23	case	8	castrated male	>1 severe	yes	H1/H1
24	case	1.5	spayed female	>1 severe	no	H1/H2
25	case	3	intact male	>1 severe	no	H1/H2
26	case	7	castrated male	>1 severe	yes	H1/H1
