# Supplementary material for: An ancient haplotype containing antimicrobial peptide gene variants is associated with severe fungal skin disease in Persian cats
Source: PLoS Genet. 2022 Feb 14;18(2):e1010062. doi: 10.1371/journal.pgen.1010062 (PMC8880935; doi:10.1371/journal.pgen.1010062)
Supplement: S2 Table — (PDF) [file pgen.1010062.s007.pdf]

S2 Table

Genomic coordinates	p-value	-log10(p-value)	Coding status and gene
F1:70627135	$1.5x10^{-10}$	9.8	noncoding
F1:70765913	$1.5x10^{-10}$	9.8	noncoding
F1:70779519	$1.5x10^{-10}$	9.8	noncoding
F1:70805614	$1.5x10^{-10}$	9.8	synonymous *ADAR*
F1:70874630	$1.5x10^{-10}$	9.8	noncoding
F1:71194608	$1.5x10^{-10}$	9.8	noncoding
F1:71418798	$1.5x10^{-10}$	9.8	noncoding
F1:71613709	$1.5x10^{-10}$	9.8	noncoding
F1:71655990	$1.5x10^{-10}$	9.8	non-synonymous *S100A9*
F1:70625102	$5.6x10^{-10}$	9.3	noncoding
F1:70663397	$5.6x10^{-10}$	9.3	noncoding
F1:70863899	$5.6x10^{-10}$	9.3	noncoding
F1:70918138	$5.6x10^{-10}$	9.3	noncoding
F1:70983681	$5.6x10^{-10}$	9.3	noncoding
F1:70995347	$5.6x10^{-10}$	9.3	noncoding
F1:70998063	$5.6x10^{-10}$	9.3	noncoding
F1:70998140	$5.6x10^{-10}$	9.3	synonymous *ATP8B2*
F1:71003152	$5.6x10^{-10}$	9.3	synonymous *ATP8B2*
F1:71024521	$5.6x10^{-10}$	9.3	noncoding
F1:71024714	$5.6x10^{-10}$	9.3	noncoding
F1:71027253	$5.6x10^{-10}$	9.3	noncoding
F1:71054850	$5.6x10^{-10}$	9.3	noncoding
F1:71055079	$5.6x10^{-10}$	9.3	noncoding
F1:71172278	$5.6x10^{-10}$	9.3	noncoding
F1:71194053	$5.6x10^{-10}$	9.3	noncoding
F1:71197407	$5.6x10^{-10}$	9.3	noncoding
F1:71266005	$5.6x10^{-10}$	9.3	noncoding
F1:71408457	$5.6x10^{-10}$	9.3	noncoding
F1:71448742	$5.6x10^{-10}$	9.3	noncoding
F1:71619500	$5.6x10^{-10}$	9.3	noncoding
F1:71643794	$5.6x10^{-10}$	9.3	noncoding
