# Supplementary material for: An ancient haplotype containing antimicrobial peptide gene variants is associated with severe fungal skin disease in Persian cats
Source: PLoS Genet. 2022 Feb 14;18(2):e1010062. doi: 10.1371/journal.pgen.1010062 (PMC8880935; doi:10.1371/journal.pgen.1010062)
Supplement: S3 Table — (PDF) [file pgen.1010062.s008.pdf]

S3 Table

Gene	Genomic coordinates	NCBI protein ID	Amino acid alteration[a]	PROVEAN score	Effect prediction	AF in domestic cats[b]	GWAS p-values[c]
IL6R	F1:70945999	XP_023103841.1	T287A	-0.357	neutral	0.303	$2.6x10^{-5}$
NUP210L	F1:71150962	XP_019678071.2	V590I	0.018	neutral	0.059	$3.5x10^{-5}$
NUP210L	F1:71168597	XP_019678071.2	S968P	-3.015	deleterious	0.067	-
NUP210L	F1:71179382	XP_019678071.2	A1358T	-0.406	neutral	0.062	-
NUP210L	F1:71179388	XP_019678071.2	R1360G	2.137	neutral	0.503	-
CREB3L4	F1:71210681	XP_003999812.1	C269R	1.989	neutral	0.054	-
CRTC2	F1:71227249	XP_003999815.3	S236A	-0.908	neutral	0.644	$1.2x10^{-2}$
CRTC2	F1:71229627	XP_003999815.3	Q517P	-1.526	neutral	0.128	-
S100A4	F1:71536030	XP_003999828.1	Y17H	4.92	neutral	0.697	-
S100A15	F1:71612744	XP_019676968.1	A43V	1.242	neutral	0.179	$5.7x10^{-7}$
S100A12	F1:71639972	XP_006943210.2	W17R	3.935	neutral	0.588	$2.3x10^{-2}$
S100A12	F1:71640026	XP_006943210.2	E35Q	1.444	neutral	0.588	$7.3x10^{-3}$
S100A12	F1:71640518	XP_006943210.2	E67G	0.245	neutral	0.590	-
S100A12	F1:71640554	XP_006943210.2	C79S	0.285	neutral	0.366	$7.3x10^{-3}$
S100A9	F1:71653999	XP_003999832.3	A109T (A87T)	2.004	neutral	0.572	$7.3x10^{-3}$
S100A9	F1:71654029	XP_003999832.3	K99E (K77E)	1.999	neutral	0.572	$7.3x10^{-3}$
S100A9	F1:71654035	XP_003999832.3	D97N (D75N)	-0.054	neutral	0.572	$7.3x10^{-3}$
S100A9	F1:71654085	XP_003999832.3	V80A (V58A)	1.045	neutral	0.585	$3.8x10^{-4}$
S100A9	F1:71654088	XP_003999832.3	T79N (T57N)	-0.029	neutral	0.585	$3.8x10^{-4}$
S100A9	F1:71654104	XP_003999832.3	E74Q (E52Q)	-0.095	neutral	0.588	$7.3x10^{-3}$
S100A9	F1:71655946	XP_003999832.3	E64K (E42K)	-0.916	neutral	0.592	$2.9x10^{-3}$
S100A9	F1:71655961	XP_003999832.3	M59L (M37L)	0.489	neutral	0.567	$2.9x10^{-3}$
S100A9	F1:71655969	XP_003999832.3	P56Q (P34Q)	3.236	neutral	0.361	$5.7x10^{-7}$
S100A9	F1:71655985	XP_003999832.3	A51P (A29P)	3.156	neutral	0.570	$2.9x10^{-3}$
S100A9	F1:71655990	XP_003999832.3	G49E (G27E)	-6.244	deleterious	0.149	$1.5x10^{-10}$
S100A9	F1:71656006	XP_003999832.3	H44Y (H22Y)	3.497	neutral	0.588	$7.3x10^{-3}$
S100A9	F1:71656065	XP_003999832.3	A24E (A2E)	-0.978	neutral	0.149	$5.7x10^{-7}$

AF, allele frequency; GWAS, genome wide association study

[a]Amino acid alterations are reported as the residue found in the FCA_H2 (control) haplotype followed by the residue in the FCA_H1 (case) haplotype. For *S100A9*, the text in parenthesis corrects for a putatively mis-annotated start site in the feline reference genome.

[b]Allele frequency (AF) in domestic cats was determined from the publicly available variant data for domestic cats described in the methods.

[c]Some markers were removed during filtering steps prior to GWAS and therefore do not have p-values; for example, F1:71168597 in *NUP210L* was filtered due to one cat lacking a genotype (100% call rate was required to be included in GWAS).
