# Supplementary material for: An ancient haplotype containing antimicrobial peptide gene variants is associated with severe fungal skin disease in Persian cats
Source: PLoS Genet. 2022 Feb 14;18(2):e1010062. doi: 10.1371/journal.pgen.1010062 (PMC8880935; doi:10.1371/journal.pgen.1010062)
Supplement: S4 Table — (PDF) [file pgen.1010062.s009.pdf]

S4 Table

Genes	Skin mRNA expression[a]	Skin-specific expression
KCNN3	Yes	No
LOC111558424	No	No
ADAR	Yes	No
CHRNB2	No	No
UBE2Q1	Yes	No
SHE	Yes	No
IL6R	Yes	No
ATP8B2	Yes	No
AQP10	No	No
HAX1	Yes	No
UBAP2L	Yes	No
CF1H1orf43	Yes	No
CF1H1orf189	No	No
TPM3	Yes	No
NUP210L	No	No
RPS27	Yes	No
RAB13	Yes	No
JTB	Yes	No
CREB3L4	Yes	No
SLC39A1	Yes	No
CRTC2	Yes	No
DENND4B	Yes	No
GATAD2B	Yes	No
SLC27A3	Yes	No
INTS3	Yes	No
NPR1	Yes	No
ILF2	Yes	No
SNAPIN	Yes	No
CHTOP	Yes	No
S100A1	Yes	No
S100A13	Yes	No
S100A14	Yes	No
S100A16	Yes	No
S100A2	Yes	No
S100A3	Yes	No
S100A4	Yes	No
S100A5	No	No
S100A6	Yes	No
LOC101087705 (*S100A15*)	Yes	Yes
S100A8	Yes	No
S100A12	Yes	No
S100A9	Yes	No

[a]RNA-seq data for various feline tissues accessed via NCBI's Genome Data Viewer for genome assembly Felis_catus_9.0 (GCF_000181335.3).
