# Supplementary material for: An ancient haplotype containing antimicrobial peptide gene variants is associated with severe fungal skin disease in Persian cats
Source: PLoS Genet. 2022 Feb 14;18(2):e1010062. doi: 10.1371/journal.pgen.1010062 (PMC8880935; doi:10.1371/journal.pgen.1010062)
Supplement: S5 Table — (PDF) [file pgen.1010062.s010.pdf]

S5 Table

**Common Name**	**Scientific Name**	**NCBI Accession Numbers**
Asiatic wild cat	*Felis silvestris ornata*	SRR15116525 & SRR15116526 & SRR7621238
Chinese desert cat	*Felis silvestris bieti*	SRR7621229
Sand cat	*Felis margarita*	SRR12385451
Black-footed cat	*Felis nigripes*	SRR2511864
Jungle cat	*Felis chaus*	SRR2062187
Flat-headed cat	*Prionailurus planiceps*	SRR6071639
Rusty-spotted cat	*Prionailurus rubiginosus*	SRR6071640
Fishing cat	*Prionailurus viverrinus*	SRR6071638
Asian leopard cat	*Prionailurus bengalensis*	GCA_016509475.1
Pallas' cat	*Otocolobus manul*	SRR5043308
Cheetah	*Acinonyx jubatus*	SRR2737519 & SRR2737521
Asian golden cat	*Pardofelis temminckii*	SRR6071641
Bobcat	*Lynx rufus*	SRR6071633
Eurasian lynx	*Lynx lynx*	ERR1255552
Canada lynx	*Lynx canadensis*	SRR6071634
Pampas cat	*Leopardus colocolo*	SRR6071642
Geoffroy's cat	*Oncifelis geoffroyi*	GCA_018350155.1
Oncilla	*Leopardus tigrinus*	SRR6071644
Serval	*Profelis serval*	SRR6071636
Caracal	*Profelis caracal*	SRR12378069
Lion	*Panthera leo*	GCA_018350215.1
Tiger	*Panthera tigris*	GCA_018350195.1
